# “Caring for Breast Cancer Patients Is a Work That Brings Sorrow”: A Qualitative Interview Study with Nurses in Tanzania

**DOI:** 10.1177/23779608231187241

**Published:** 2023-07-10

**Authors:** Paulo L. Kidayi, Hélio Manhica, Sandra Nakazzi, Christina C. Mtuya, Ragnhild E. Aune, Furaha Serventi, Michael Johnson Mahande, Gunilla Björling

**Affiliations:** 1 Faculty of Nursing, 108094 Kilimanjaro Christian Medical University College, Moshi, Tanzania; 2 Department of Global Public Health, 27106 Karolinska Institutet, Stockholm, Sweden; 3 Department of Health Promotion, Sopiahemmet University, Stockholm, Sweden; 4 Department of Neurobiology, Care Sciences and Society, 27106 Karolinska Institutet, Stockholm, Sweden; 5 Department of Material Sciences, 8018 Norwegian University of Technology and Science, Trondheim, Norway; 6 Kilimanjaro Christian Medical Centre Cancer Care Centre, Moshi, Tanzania; 7 Institute of Public Health, 108094 Kilimanjaro Christian Medical University College, Moshi, Tanzania; 8 Department of Nursing, School of Health and Welfare, 4161 Jönköping University, Jönköping, Sweden

**Keywords:** nurses, experience, caring, breast cancer, oncology

## Abstract

**Introduction:**

Breast cancer is increasing in sub-Saharan Africa (SSA), and most women are diagnosed at a late stage. This leads to increased suffering for the patients and challenging care situations for nurses. Limited resources in healthcare, lack of oncology training, and low health literacy in society result in even more demanding situations for nurses.

**Objective:**

The objective was to explore nurses’ experiences of caring women for with breast cancer in Tanzania.

**Methods:**

The study employed a descriptive qualitative design. Fifteen nurses, working in oncology units in three major hospitals in Tanzania were interviewed using a semistructured interview guide. The participants had a minimum of 6 months experience of caring for breast cancer patients. Purposive sampling was used. Data were analyzed by qualitative content analysis.

**Results:**

Two main themes emerged: *Challenges in caring for breast cancer patients* and *Nurses’ psychological distress*. The late diagnosis was very challenging for the nurses. Low health literacy regarding breast cancer disease and treatment, patients’ financial difficulties, minimal oncology nursing education, and technology in healthcare systems were also major challenges. The nurses experienced psychological distress, lost hope, and faced ethical dilemmas while providing cancer care.

**Conclusion:**

The findings of this study conclude that nurses face emotional distress and ethical dilemmas while caring for patients with breast cancer. Late diagnosis, lack of infrastructure and resources, and low health literacy among patients, family, and healthcare providers have a great impact on the stress that the nurses experience.

## Introduction

Breast cancer is an emerging burden in low-income countries (LIC), including Tanzania (Bray et al., 2018; WHO, 2023). Most patients in Tanzania are diagnosed late, in stadium III or IV (Rick et al., 2021; United Republic of Tanzania, 2020; WHO, 2023). This decreases the survival rate, increases the burden of care for families, and challenges nursing care, as there are scarce resources and few cancer care centers (Kohi et al., 2019). There is as well limited education in oncology care for nurses in Tanzania, which jeopardizes the caring situation for the nurses and the patients.

### Review of Literature

Globally, the incidence of cancer is estimated to be between 19.3 million and 10.0 million projected to reach 28.4 million cases in 2040 (Sung et al., 2021). Breast cancer accounts for about 11.7% of the total cancer cases. In Tanzania, the annual cancer incidence is estimated to be 40,000 cases yearly whereof 80% die within 5 years. Breast cancer is the second most common cancer after cervical cancer, with a high mortality rate (United Republic of Tanzania, 2013, 2023). Also, about 80–90% of cancer patients are incapable to access diagnostic and healthcare facilities (United Republic of Tanzania, 2013) due to distance and poverty (Kohi et al., 2019; Rick et al., 2021; United Republic of Tanzania, 2020). In addition, 70% of the cancer patients did not take the prescribed anticancer drugs (Yohana et al., 2011). Despite the government efforts to improve healthcare services, human resources for health, including nurses in Tanzania, remained a major problem with a shortage of staff of 50% national wise (United Republic of Tanzania, 2023), which has a great impact on nurses’ work environment. There is as well a lack of cancer care at the primary healthcare level (Brand et al., 2019; United Republic of Tanzania, 2013), rendering late diagnosis (Rick et al., 2021). Inequalities in the accessibility of health services, including cancer services, were found to expose nurses to many stressors, particularly when patients present late with severe illness (Maree & Mulonda, 2017a).

A systematic review of the prevalence of burnout in oncology nurses showed that there is a considerable risk for burnout, as the level of emotional exhaustion among oncology nurses is high (Cañadas-De la Fuente et al., 2018). Moreover, a study from China (Guo & Zheng, 2019) showed that most oncology nurses (73–86%) experienced moderate to high levels of burnout, caused by challenges in caring for palliative cancer patients. Similarly, nurses in Nigeria expressed being affected mentally by frequent encounters with patients dying from cancer (Nwozichi et al., 2020). These experiences are accelerated by limited oncology training among nurses in sub-Saharan Africa (SSA) (Jenkins et al., 2020). Furthermore, the lack of oncology education also affects the nurses negatively and the need for oncology nursing education in SSA is evident (Nwozichi et al., 2017). With low education in oncology, nurses are less likely to cope with the psychological pressure when caring for cancer patients, thus prone to emotional and psychological stress (Nwozichi et al., 2020). With inadequate human resources for health as in SSA, breast cancer patients face challenges in terms of disease progression and therapeutic side effects for their entire life.

In Tanzania, like in other countries in SSA, i.e., Nigeria, (Nwozichi et al., 2017), there is only one master of science program in oncology nursing with limited enrolment. Thus, cancer care is often given by nononcology-trained nurses who face huge challenges when caring for this patient group. The lack of knowledge of cancer care among nurses could affect the regional contribution to the WHO target, i.e., reducing the cancer mortality rate by 2.5% per year globally among women with breast cancer (WHO, 2023).

Given the limited access to cancer care, limited infrastructure, and lack of resources and oncology nursing training in Tanzania, the work situation for oncology nurses is very demanding. To improve the situation and develop oncology education, it is important to conduct more research in the field. Therefore, the purpose of this study was to explore nurses’ experiences in caring for patients with breast cancer in Tanzania.

## Methods

### Design

This descriptive qualitative study used an empirical inductive approach by Polit and Beck (Polit & Beck, 2017). Semistructured interviews were performed with fifteen nurses, and a content analysis method was used (Graneheim & Lundman, 2004). The study analysis and writing follow the consolidated criteria for reporting qualitative studies (COREQ) checklist for qualitative research (Tong et al., 2007).

### Research Question

What are nurses’ experiences of caring for women with breast cancer in Tanzania?

### Sample

The study was conducted in three major hospitals in Tanzania that provide oncology services: Kilimanjaro Christian Medical Centre (KCMC) in Moshi, northern Tanzania, Bugando Medical Centre (BMC) in Mwanza, western Tanzania, and Ocean Road Cancer Institute (ORCI) in Dar es Salaam, in eastern Tanzania. The distance between KCMC and BMC is 843 km, and between KCMC and ORCI, it is 548 km. Between BMC and ORCI, it is 1134 km. In total, 110 nurses work in cancer care centers; 15 in KCMC, 15 in BMC, and 80 in ORCI.

The participants consisted of registered nurses employed at oncology units in these hospitals. A purposive sampling method was employed to select a minimum of five participants at each selected hospital. We planned to interview at least 15 nurses in total; however, the saturation point of the data was reached at 12 participants and three more interviews (already planned) were conducted, one at each hospital, to confirm the data saturation (Polit & Beck, 2017).

Firstly, the head nurse of the oncology unit at the respective hospital was contacted and informed about the study. Thereafter, the first author approached the nurses during their normal shifts at each study site, informed them about the study, and invited them to participate. All recruited nurses agreed to participate, and the interviews were completed in the last hour of the work shift. All participants gave their written informed consent before the interview.

### Inclusion/Exclusion Criteria

Inclusion criteria were registered nurses, with a working experience of at least 6 months in caring for patients with breast cancer at oncology units.

Exclusion criteria were nurses, who did not want to participate in the study, nurses who had less than 6 months of experience in oncology care, and nurses from other hospitals who were visiting the oncology units.

### Institutional Review Board Approval

The study was performed following the Declaration of Helsinki. Ethical clearance certificates were obtained from Kilimanjaro Christian Medical University College, No 2488 Ethics Committee and from the National Institute for medical research in Tanzania, NIMR/HQ/R.8a/Vol.IX/3904. Permission letters were obtained from respective Regional Medical Officers, and permission was sought and obtained from the respective hospital. Moreover, written informed consent was obtained from the participants before the interview started. Participation was voluntary, and the participants could withdraw at any time without explanation. All data were anonymized to maintain confidentiality during the study.

### Data Collection

We used face-to-face semistructured interviews with registered nurses working in both inpatient and outpatient departments in three cancer care centers in Tanzania. A semistructured interview guide was used, based on the research questions of the study (Polit & Beck, 2017). The interview guide was pretested in two pilot interviews in two different oncology units, to test and adjust the guide and ensure its validity before being used. However, there was no need for adjustment of the interview guide, and the guide remained the same during the entire data collection which ascertained the topics covered in all interviews (Graneheim & Lundman, 2004). Pilot interviews are recommended to test the interview guide (Polit & Beck, 2017). The interviews took place at the end of the work shift within the oncology units in a selected place convenient for the participants to guarantee the privacy of their information and lasted from 40–60 min. The interview guide was followed to ascertain that the topics were covered in all interviews, but the interviewer strived to be open to the stories told by the participants so that no critical topics/experiences were missed. Each interview was recorded with the permission of the participants, and field notes were taken from each participant by an independent notetaker during the interview process. All interviews were transcribed verbatim within hours upon completion of each of the interview sessions to preserve their original meaning (Graneheim & Lundman, 2004). The interviews were conducted in Kiswahili and translated into English by a person who was fluent in both languages.

### Data Analysis

The interview responses were analyzed with qualitative content analysis, by using the principles of Graneheim and Lundman (2004) to identify prominent themes and patterns in the text. Qualitative content analysis should be appropriate even when knowledge seems to be intrinsically captured or generally designed (Graneheim et al., 2017; Lincoln & Guba, 1985). Firstly, the interviews were transcribed verbatim, and to ensure and obtain the quality of data, the researchers read through several times to reach a deeper understanding of the transcribed texts and ensured the validity of data accumulated to identify patterns out of the information provided by the informants. The analysis process involved breaking down data into smaller units, coding, and naming the units according to the content in the text. Thereafter, the units were categorized based on the shared concepts to identify prominent themes and patterns in the transcribed text. An inductive approach was used during the analysis phase, such that the researchers could find the likeness and contrast in the data, and thereafter, the information was characterized by formulating themes and subthemes that depended on the researchers’ interpretation and abstract that seemed to answer the research questions formed for the study and discussed among three researchers (Graneheim & Lundman, 2004).

To minimize the risk of implication on the researchers’ interpretations of data during the analysis phase, four data saturation criteria were applied: credibility; dependability; transferability, and confirmability, which are vigorously recommended for qualitative research to strengthen the findings of the study (Lincoln & Guba, 1985; Polit & Beck, 2017).

Credibility was enhanced as we used well-established research methods in the study and that data were gathered from the primary source of nurses working at the three national cancer care centers.

Trustworthiness was ensured as data were derived from nondependable participants and the researcher who performed the interviews followed the interview guide and had no previous relation to the informants. Furthermore, the data analysis was guided by content analysis and three independent researchers analyzed the data with varied interpretations accelerating transparency of the findings derived from the original data, which also increased the dependability of the study and ensured the confirmability of the study. Moreover, the transferability was enhanced by describing the data collectors, the collection of data, the duration of the interviews, and how the analysis was performed. Thereby, the study can be transferred to other settings (Lincoln & Guba, 1985; Polit & Beck, 2017).

## Results

### Sample Characteristics

In total, 15 registered nurses participated in the study, whereof 11 were female and 4 were male. Their age ranged from 24 to 56 years and the experience of caring for patients with breast cancer ranged from 1 to 16 years. Six nurses had not attended any course in oncology, while the majority had some prior form of oncology training, however, mostly in the form of short courses. Sociodemographic data are presented in Table 1.

**Table 1.** Sample Characteristics.

Respondent	Age (years)	Sex	Experience of cancer care (years)	Experience as a nurse (years)	Special training on oncology
P1	43	F	1	20	Postgraduate diploma in palliative care. Medicine and basic training on safe handling of chemotherapy
P2	43	F	2	9	Training on pediatric oncology
P3	52	F	10	25	Three days training of chemotherapy for children and adults. Palliative care
P4	24	M	3	3	None
P5	36	F	3	8	Hematology and pediatric oncology. Safe handling of cytotoxic drugs
P6	38	F	9	11	Introduction to oncology disease and treatment
P7	43	F	7	15	None; continuous medical and nursing education at the working area
P8	43	F	16	16	Two weeks, oncology in general, radiotherapy, care of wound and palliative care
P9	47	M	16	16	Two days, breast and cervical cancer screening
P10	45	M	16	16	One-week oncology in general and continuous education in the working area
P11	43	F	7	15	Three months; Tanzania: Breast and cervical cancer screening. Outside Tanzania: Care of patients with Cancer and administration of chemotherapy
P12	28	M	2	5	None
P13	35	F	3	5	None
P14	56	F	4	24	None
P15	29	F	5	5	None

### Research Questions Results

The content analysis resulted in two main themes and eight subthemes. The main themes are *Challenges in caring for women with breast cancer* and *Nurses’ psychological distress*. The main themes and subthemes are presented in Figure 1.

**Figrue 1.**
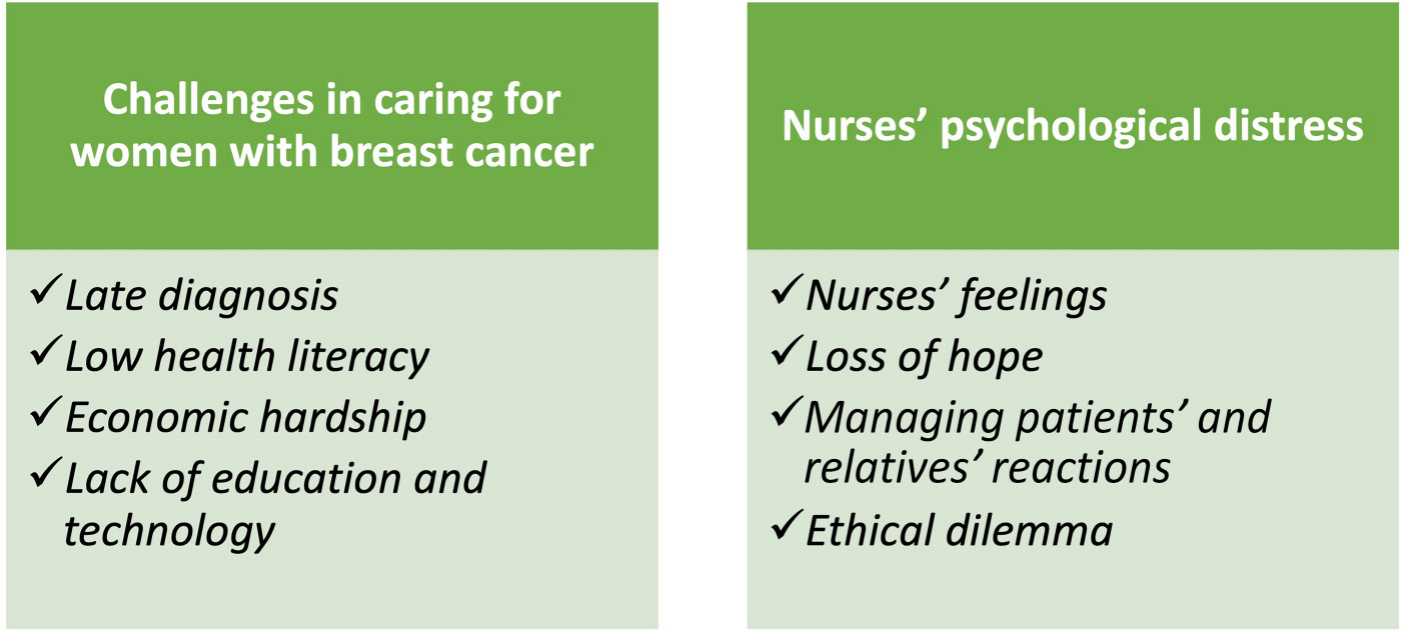
Composed themes and subthemes.

### Challenges in Caring for Women with Breast Cancer

This theme includes different challenges that affect nursing care. The nurses narrated that meeting so many patients in advanced stages of breast cancer is very challenging. Many women also seem to lose self-confidence and autonomy after mastectomy, because they do not see themselves as complete and their self-esteem is strongly affected. This was difficult to handle for the nurses. Dealing with stigma and misbelief is an enormous task for the informants, especially when involving family members with misconceptions and traditional beliefs.

#### Late Diagnosis

One of the biggest challenges for all the nurses in the study was when patients are seeking help for cancer at a late stage. Most women seek healthcare very late for a problem that has lasted for a very long time without medical attention. The reason could be that they are afraid of the expensive medical bills and that they might not be able to afford the treatment; hence many women turn to herbal medications for relief and not for cure:

They are coming at stage four. It is a big challenge we have. They use herbal medicines, sometimes they mix, miss follow ups and …sometimes you might find the patient receiving chemotherapy, but they do not come to the clinic (follow up).

Several nurses narrated that when healthcare providers notice the problem on time, patients usually start their treatment instantly to increase their chances of cure and survival. Others can come when it is already at stage four, which requires either mastectomy or palliative care. Many nurses reported that most women with breast cancer seek medical attention very late and the chance of cure is, therefore, extremely low.

…Some of our patients come to the hospital in an advanced stage of disease, why you’ll find that cancer has spread…with metastases…

I see a need for education, I hope breast cancer education should be given widely so that it can be identified early because most of the patients come at stage three or later. But to identify patients at early stage…, it is very difficult.

#### Low Health Literacy

Lack of knowledge, such as understanding and using health information for self-management in seeking or utilizing healthcare services, has a huge impact on the quality of care for a patient with breast cancer. The nurses reported that breast cancer patients and their relatives face major challenges in understanding and caring for themselves during their disease. Families also lack adequate information or awareness regarding cancer.

Low health literacy on cancer is a problem in the community. Patients face difficulties while seeking treatment for minor health problems, not related to their cancer, within primary care, as nurses and clinical officers refuse to give them any services once they find it is a cancer patient. The patients are, therefore, advised to consult the oncology clinic regardless of the type of care service they are seeking. Either the patients return home, where the condition might worsen, or decide to go to the oncology clinic with minor problems which will lead to unnecessary costs due to, e.g., distance. Furthermore, the nurses narrated that patients are affected emotionally, not due to the disease or cancer treatment but due to being isolated by family members due to the cancer diagnosis and are treated like they have been cursed. They told the nurses that their families and/or husbands ignore them:

Many are left by their husbands. Others, whenever they go to their relatives, the relatives are not helping. People don’t accept breast cancer as a problem like other diseases……. they think, once you get breast cancer you’re destined for death.

The nurses described that low health literacy leads to misconceptions among breast cancer patients and their relatives, which delays the diagnosis. Some persons believe cancer is incurable and that once acquired, one will die. The nurses described that patients choose to go to traditional healers, as they believe themselves being bewitched or cursed. This misunderstanding makes both patients and relatives neglect formal medical treatment in hospitals. However, while being treated by traditional healers and the condition becomes worse, patients decide to seek hospital care, which usually happens at a late disease stage:

First, she believes, there are no drugs to assist her. Once herbal medicine is taken the disease spreads quickly. She thinks she is destined for death only. When patients are told, they are supposed to start chemotherapy the patients say, - Give me two months.

#### Economic Hardship

Nurses described that poverty, cost sharing, and shortage of resources were the major challenges in providing effective cancer care service. Despite improved infrastructure at the hospitals, the nurses narrated that, in both public and private hospitals, cancer drugs sometimes are out of stock. Regardless of whether a patient has the means to buy drugs, it is not easy to get hold of the drugs in a normal pharmacy leading to discontinuation of treatment.

When drugs are out of stock, you can’t get them anywhere and the treatment is, therefore, disrupted. Patients must wait until the drugs are available.

Patients often must stop the recommended treatment due to high medication costs and their financial situation. Consequently, they do not show up for appointments scheduled for the next dose of chemotherapy, or for other essential follow-ups. The patients’ incapability to afford the treatment, which sometimes, already might have been bought by the hospital, can lead to insolvency in running the cancer centers and affects the payment of healthcare staff.

We buy chemotherapy, and as you know chemotherapy is very expensive. The patient comes here and leaves no money, and the patient complains about money, so, we are doing this as a team: doctors, nurses, and social workers to help the patient with the chemotherapy.

The need to attain health insurance from the National Health Insurance Fund (NHIF) in Tanzania, to get free access to medical services, is a great challenge for the patients at the cancer centers and affects the nurses’ situation. Nurses also observed that economic challenges contribute to a difficult caring environment for interactions between the patients and staff, as no one is comfortable. Nurses are overworked due to a shortage of equipment and shortage of staff and hence fail to maintain good practice, and patients fail to pay for their service which contributes to poor health-seeking behavior:

…..Yes, we are working beyond the national and international standards of nursing practice, for example, you may find two nurses giving chemotherapy to 20 or 35 patients. Sometimes patients are dissatisfied as they may need help, but you don’t have time to listen to them.

#### Lack of Education and Technology

The nurses described that, in general, they had low knowledge and skills in caring for breast cancer patients, despite attending short courses, medical and nursing continuing education at their institutions. Some of them did not have any training in oncology care at all. The curriculum, both diploma- and bachelor's degree nursing programs do not have enough content for oncology to enable nurses to obtain knowledge and skills to care for cancer patients. The nurses acknowledged that there was not any nurse specialist in oncology care in all three cancer care centers:

When I started, I had so many deficits. You know, you don’t have the knowledge about complications. …. -You can’t educate the patient well.

The nurses also described that technology influences health services. Hospitals use electronic systems for registry, payment, clerking, and documentation of all services provided to patients. Low digital literacy among nurses contributes to disturbances in care, like inaccurate recording of patients’ information. Furthermore, internet and electricity fluctuations (cut off) contribute to a delay in providing health services to the patients, as the nurses fail to give services without support from the internet and electronic medical records. There is as well an inaccurate recording of information in the system, especially when the healthcare providers fail to enter the patients’ information.

… You gave chemotherapy, then finished in the morning, the network fails during the night… you documented in the system, but the time that it will appear there, it is not in the system

### Nurses’ Psychological Distress

This theme refers to various forms of subjective periods of anguish or emotional states related to the working environment, as well as to communicate with patients and relatives. The nurses described that caring for breast cancer patients sometimes is very distressing as the majority are diagnosed late and have incurable conditions, while others refuse to take a biopsy for further breast cancer investigations and some reject treatment with chemotherapy. The ethical dilemmas that nurses face also affect the nurses.

#### Nurses’ Feelings

Patients, who come late to healthcare facilities with advanced diseases affect the nurses emotionally. The interviewed nurses narrated that, with their knowledge of disease and treatment, they keep in mind that the patient will probably die, and when they are at home, after work, they still think of the patients and feel for their conditions. Despite the stressful situation for the nurses, they are not offered any psychological support like counseling to keep their mental health intact:

Caring breast cancer patients… sometimes, I feel bad, especially for patients who come late. They come with a big wound, with severe pain, sometimes oozing with pus, or with a fungating tumor. I feel also very bad when a patient comes late and has metastases. Metastasis in the lungs, giving difficulty in breathing and their chest x-ray shows pleural effusion, but they refuse to have breast biopsies taken to confirm the diagnosis. I feel very bad.

#### Loss of Hope

The nurses also narrated that the breast for a woman reflects her beauty, reproductive, and child-nurturing nature. Therefore, the removal of a woman's breast results in sorrow. Many patients also lose hope regarding breast cancer services due to misconceptions or traditional beliefs about cancer diagnosis and treatment. Moreover, nurses get emotionally involved and tend to be part of the patients’ loss of hope, as most patients come at a late stage with big wounds and metastasis and end up being scheduled for palliative care. This promotes the misconception and traditional belief in the community that breast cancer is incurable. Moreover, there is stigma and discrimination, which influences the patient attendance to treatment, and the nurses lose hope, in themselves, and are sad and distressed when they see patients being abandoned by their family members:

…When you see that the person is suffering and at the end of the day you know she will die. We are putting in effort, but you know that the patient will die, since some patients come at the late stages, it hurts. You place yourself into the shoes of the patient or relative… it is torture, somehow, until you get experienced, but still, it hurts.

#### Managing Patients’ and Relatives’ Reactions

Families and patients show different reactions once healthcare providers disclose the diagnosis and treatment for them. The nurses said that some relatives ask healthcare staff to withhold results from the patients because they might die or reject the formal cancer treatment and seek alternative treatment options like traditional medicinal herbs. This problem leads to psychological distress for nurses, patients, and relatives:

… Imagine, the patients diagnosed with cancer, some go home and never return. Some say, -wait, I am going for a prayer. When she comes back, the breast cancer has reached stage IV and metastasized. Others say, - leave me, I don’t want to be treated.

This is frustrating to the nurses as they keep thinking that the patient's condition will get worse. Even if she will come back later, she will be scheduled for palliative care or premature death may occur, when the patient might have been cured:

For example, one client refused to take a biopsy for diagnosis. She wanted to go for prayer because she had been told that, once you take a biopsy immediately metastasis starts. Another died for the same reason. There are so many, who say, - Leave me.

Nurses also narrated that the reactions from relatives varied, and so did their attitudes to the patients. Those with a positive attitude assist their sick relative. Relatives with negative attitudes tend to persuade their sick relatives to reject cancer services; hence this acts as a barrier to cancer service both in clinical and community settings.

#### Ethical Dilemma

The nurses described pain from big wounds with pungent smells as one factor that keeps patients uncomfortable, especially when they meet other patients or healthcare staff. Some patients and relatives believe that cancer is incurable, which is difficult for nurses. The late condition with big wounds and metastases frustrates patients and relatives and puts the nurses in an ethically difficult situation. Sometimes the patients scheduled for chemotherapy ask the nurse whether they will be cured or not. The nurses cannot answer them truthfully without hurting the patients:

Caring for breast cancer patients is a work that brings sorrow. The patients I meet are those who come late. Even if you are giving service to patients, you don’t see any hope for the future. When she asks, - if I get these drugs will I be cured? You have nothing to say. You remain in a dilemma.

The shortage of nurses in the oncology units also left the nurses in a difficult situation as the time for each patient is limited. This was frustrating for the nurses as they knew the patients needed time for care and comfort and the nurses could not be there for them.

## Discussion

This study aimed to determine the experiences of nurses in caring for women with breast cancer in Tanzania. Two main themes emerged: *Challenges in caring for breast cancer patients* and *Nurses’ emotional stress*. The findings show that cancer care is very challenging for nurses and brings sorrow and emotional stress. Contributing factors were late diagnosis, low health literacy with traditional beliefs and misconceptions, and poverty. It is important to improve health literacy on cancer to avoid late diagnosis and to reduce the burden for patients, families, and nurses in oncology care.

### Challenges in Caring for Breast Cancer Patients

#### Late Diagnosis

The findings of the study revealed that there are many challenges for the nurses in Tanzania who care for women with breast cancer, and the biggest challenge was encountering patients who are diagnosed late. The late diagnosis of cancer is a major problem in LIC (Brand et al., 2019), including Tanzania, where most women with breast cancer are diagnosed with stage III or IV (Sung et al., 2021; United Republic of Tanzania, 2023). Encountering and caring for patients with a late cancer diagnosis is very stressful for nurses, as previous research show (Guo & Zheng, 2019; Nwozichi et al., 2020). Our findings emphasize how big this challenge is for the nurses, especially in combination with low health literacy among patients and families, as well as the limited economic resources for both patients and in healthcare settings. Therefore, it arouses an immediate awareness of interventions to rectify the misperception about breast cancer and also to provide clear guidance on the screening programs for breast cancer. Breast awareness provides women with knowledge on how to detect breast diseases and reduces morbidity (McCready et al., 2005). To decrease the number of women with advanced breast cancer, outreach programs to conduct screening of women in the community need to be prioritized in Tanzania (Rick et al., 2021).

#### Low Health Literacy

The findings show that a cancer diagnosis is a huge burden for both patients and their families and renders difficult care situations also for the nurses, particularly, as most patients and families have low health literacy and know little about cancer. Our findings are supported by research where low health literacy is pointed out as the primary cause of late diagnosis and neglect of symptoms (Rick et al., 2021). Moreover, economic hardship, particularly financial difficulties of patients and relatives (Getachew et al., 2020; Ogunkorode et al., 2021), contributes to patients’ late diagnosis and discontinuity of treatment, as patients and their relatives fail to afford treatment costs and turn to traditional medicine. Furthermore, poverty, ill-equipped healthcare, few centers for cancer care, lack of resources for healthcare, and scarcity of high-quality infrastructure contribute in LIC to inadequate treatment of most patients with breast cancer (Rivera-Franco & Leon-Rodriguez, 2018). All these factors put an additional burden on nurses when caring for breast cancer patients, which is confirmed by research from Zambia (Maree & Mulonda, 2017b).

#### Economic Hardship

The economic hardship is very worrying for the patients, and sometimes they fail to present at treatment due to financial difficulties. In a study on adolescents with cancer in Tanzania, financial difficulties were found to be a large concern for the patients and their families (Kohi et al., 2019). Due to poverty, there is a constraint of resources and high costs for chemotherapy which hinder the success of treatment, which is frustrating for the nurses.

#### Lack of Education and Technology

Most nurses had only short education or no education in oncology nursing, which raises a concern about the need for capacity building. It is shown that nurses need to gain more confidence in their role of care and administration of oncology care, since they are frequently engaged in the patient's health condition which calls for more responsibility for the patient's safety (Ridge et al., 2018).

In addition, knowledge and clinical skills among nurses acted as barriers for nurses to deliver quality care to the patients, which also was found in a study from Ethiopia (Getachew et al., 2020). This has implication, as most nurses in the present study did not have longer oncology nursing training. The implementation of capacity building for nurses will add value to their services, as patients will receive the care they need (Nwozichi et al., 2017). Increased knowledge among nurses on oncology care will decrease misconceptions and traditional beliefs among patients and relatives since they will receive adequate information regarding breast cancer and treatment thus reducing stigma and discrimination (Ogunkorode et al., 2021). Furthermore, the use of technology and informatics in healthcare is essential for nurses, and if technology fails, it has a great impact on the care of patients. The nurses in the present study experienced frustration when the technology did not work.

### Nurses’ Psychological Distress

#### Nurses’ Feelings and Loss of Hope

The findings from this study showed that nurses were emotionally affected when caring for women with breast cancer. The findings are in line with the findings of a review, where oncology nurses were found to suffer from emotional exhaustion (Cañadas-De la Fuente et al., 2018). The nurses in our study were affected when patients presented at the cancer clinic at a late stage of disease, which is supported by other research (Rivera-Franco & Leon-Rodriguez, 2018). In addition, many patients were abandoned by their relatives due to misbelieves and stigmatization regarding cancer. This was also very difficult for the nurses, and they lost hope for the patients. These findings are also supported by other studies from palliative care (Guo & Zheng, 2019; Maree & Mulonda, 2017a; Nwozichi et al., 2020).

#### Managing Patients’ and Relatives’ Reactions and Ethical Dilemma

Another challenge for the nurses was to manage the patients’ and families’ reactions to the diagnosis. Some relatives did not want the patient to know about the disease, as the patient might die or planned to go for traditional medicine instead of undergoing medical treatment at the cancer center. The use of traditional medicine is widespread, not only because of the costs but also due to a strong traditional belief (Munkombwe et al., 2020; Ogunkorode et al., 2021). This problem leads to psychological distress for nurses, patients, and relatives.

Meeting the patients at a late stage and administrating chemotherapy was difficult for the nurses, as they knew the patient would suffer from side effects. This was especially difficult when the patient asked them if the treatment would cure them. The nurses knew that the chemotherapy prolonged the life of the patients but that the patient would not be cured. Thus, it was difficult for the nurses to answer. These kinds of ethical dilemma point toward a great need for education in oncology care, which in turn will give the nurses tools to handle similar future situations. Increased knowledge will help the nurses to deliver quality healthcare to patients with breast cancer (Nwozichi et al., 2017).

### Strengths and Limitations

The trustworthiness of the study is good as the data were collected from nondependable participants and the study settings consisted of the major cancer care centers in the country. However, no experience from nurses working with breast cancer patients scheduled for palliative care in the community was collected. However, there is no structured palliative care in the community, why we could not retrieve such participants. Furthermore, the qualitative method with semistructured interviews restricts for inclusion of many participants in the study, which might affect the trustworthiness of the study compared to studies using structured data collection. However, the study was done by using well-known research methods for qualitative research to minimize the risk. The researchers who performed the interviews followed the interview guide and had no previous relation to the informants. The confirmability was achieved as the researcher collected data independently and one independent individual performed data transcriptions and translations. Furthermore, three independent researchers with vast knowledge of qualitative methods analyzed and agreed on the themes and subthemes to be the final findings, which enhanced the transparency and obtaining valid findings from this study (Graneheim & Lundman, 2004; Lincoln & Guba, 1985). Moreover, the transferability (Graneheim & Lundman, 2004; Lincoln & Guba, 1985) was enhanced by describing the data collectors, the collection of data, the duration of the interviews, and how the analysis was performed. Thereby, the study can be transferred to other similar settings and the findings can have implications in other LIC.

### Implications for Practice

This study shows that there is an urgent need to address the situation for nurses working in oncology care regarding stress management and enhanced knowledge in oncology care. Nurses experience psychological distress while caring for patients with breast cancer which needs to be addressed and monitored. Furthermore, capacity building in oncology care for nurses in all care settings, primary care included, is very important. Enhanced knowledge of oncology care among nurses will increase patient safety, improve the low health literacy in the community, and lead to earlier diagnosis of breast cancer. Increasing the number of trained human resources by establishing oncology nursing training programs will improve the quality of cancer care, survival, and quality of life of the patients as well. In addition, the cancer centers have limited infrastructure and supplies in addition to the shortage of staff. The findings of the study can be used to inform political leaders to expand cancer services in hospitals and primary healthcare settings.

## Conclusions

The findings of this study conclude that nurses face emotional distress and ethical dilemmas while caring for patients with breast cancer. Late diagnosis, lack of infrastructure and resources, as well as low health literacy among patients, family, and healthcare providers have a great impact on the stress that the nurses experience.

Providing oncology care is very stressful and challenging for the nurses, especially for those without or with little oncology training. Therefore, there is an urgent need for institutions to address the care challenges in oncology to improve cancer services to decrease nurses’ psychological distress. Improving the capacity building for nurses through oncology training programs is essential to help the nurses to provide sustainable oncology care. Only by enhanced knowledge, women can be diagnosed earlier and thus increase their survival and quality of life.
